# A field based evaluation of adverse events following MenAfriVac^®^vaccine delivered in a controlled temperature chain (CTC) approach in Benin

**DOI:** 10.11604/pamj.2014.18.344.3975

**Published:** 2014-08-28

**Authors:** Christoph Steffen, Evariste Tokplonou, Philippe Jaillard, Roger Dia, Marie N'Deye Bassabi Alladji, Bradford Gessner

**Affiliations:** 1 Agence de Médecine Preventive, Bureau de Liaison, Ferney-Voltaire, France; 2 Agence Nationale de la Vaccination et des Soins de Santé Primaire, Ministère de la Santé du Bénin, Cotonou, Bénin; 3 Agence de Médecine Preventive, Cotonou, Bénin

**Keywords:** Neisseria meningitidis A, Meningococcus, Meningitis belt, Meningococcal conjugate vaccine, MenAfriVac, Benin, Mass vaccination, Controlled temperature chain, Pharmacovigilance, adverse events following immunization (AEFI)

## Abstract

**Introduction:**

An estimated one hundred million African meningitis belt residents have received MenAfriVac^®^meningococcal serogroup A conjugate vaccine. Since October 2012 the vaccine has been licensed for use in a controlled temperature chain (CTC) approach, at temperatures of up to 40°C for up to four days. The Benin Ministry of Health conducted a pilot evaluation in one of its 34 health districts to assess whether the CTC approach was associated with increased adverse events following immunisation (AEFIs).

**Methodes:**

We compared the occurrence of AEFIs during the 5 days following immunisation for 4 villages in the district using the CTC approach to 4 villages in another district using the traditional approach (vaccine kept at +2 to +8°C). Severe events resulting in hospitalisation or death of non-interviewed household members also were recorded.

**Results:**

We included 1000 persons in the CTC and 999 in the non-CTC group. Only mild and transient AEFIs were noted in both groups, such as pain at injection site or fever. Compared to the non-CTC group, the CTC group had similar or lower rates of AEFIs and the occurrence of AEFIs in both groups was similar to that indicated in the vaccine package insert. No case of hospitalisation or death occurred among interviewed and non-interviewed household members.

**Conclusion:**

The CTC approach, as implemented in Benin, was not associated with an increased rate of adverse events in the five days following immunisation, either when compared to a concurrent non-CTC population or to previous studies.

## Introduction

MenAfriVac^®^ - manufactured by the Serum Institute of India Ltd – is a lyophilized vaccine containing 10 mcg of purified meningococcal A polysaccharide conjugated to 10 to 33 mcg of tetanus toxoid with the addition of mannitol, sucrose, and Tris(hydroxymethyl)aminomethane. It is the first *Neisseria meningitidis* serogroup A (NmA) conjugate vaccine specifically developed for use in the African meningitis belt [1]. From its introduction in late 2010 to the end of 2012 an estimated one hundred million individuals have received the vaccine [2]. The vaccine has been used in vaccination campaigns in Burkina Faso [3], Niger [4], Mali, Cameroon, Chad, Ghana, Nigeria, Senegal, Sudan and Benin. Preliminary findings suggest that the vaccine is highly effective in reducing NmA carriage [5] and disease in Africa [6]. Data also suggest that the vaccine is safe as delivered in the traditional cold chain. The MenAfriVac^®^ package insert [7] and studies in India [8] and Mali [9] reported that the most common identified side effects at one week were pain at the injection site, diarrhea, loss of appetite, and fever.

On October 26, 2012, The Drugs Controller General of India (DCGI) granted approval to license the vaccine for use in a controlled temperature chain (CTC) approach at temperatures of up to 40°C for up to four days [7], followed by WHO prequalification for this approach [10]. The CTC approach has the potential to reduce the logistical complexity of maintaining vaccines in the +2 to +8°C temperature range up to the moment of injection of the vaccine and thus increase vaccine access to remote areas [11]. Based on its review of available clinical trial safety data, the WHO Global Advisory Committee on Vaccine Safety concluded that MenAfriVac^®^ showed a favourable safety profile [12]. Moreover, pharmacovigilance activities implemented to monitor Adverse Events Following Immunisation (AEFIs) during different mass campaigns in the past two years have confirmed this safety profile under use in the traditional cold chain approach with only minor and benign effects reported [13–15]. Benin scheduled a mass MenAfriVac^®^ vaccination campaign between 15 and 25 November 2012. During the few weeks before implementation, the Benin Ministry of Health (MoH) decided to pilot test the CTC approach, the first country globally to evaluate this approach. The present evaluation, integrated into a reinforced pharmacovigilance monitoring system, was designed to assess whether the vaccine delivered in the CTC approach resulted in unexpected AEFIs. Due to logistical and budget constraints, we did not attempt to detect rare (0.1-0.01%) or very rare events (<0.01%) or benign effects that have not been described previously in the literature.

## Methods

**Ethical issues:** the Drugs Controller General of India (DCGI) granted approval to license the vaccine for use in a CTC approach. The Benin MoH determined that the current activities did not require institutional review board approval based on a) use of an on-label approach and b) its assessment that the decision to implement and conduct a quality control evaluation of the CTC approach fell within its public health authority as part of routine immunization activities (in accordance with standards set in other locations) [16]. Nevertheless, the Benin MoH agreed to obtain informed consent from participants. Consequently, after explaining briefly the evaluation to individuals in each dwelling the investigators obtained verbal consent from all participants or their caretakers (verbal consent was obtained because of high levels of illiteracy in Benin, including the target district). As part of routine national pharmacovigilance activities, investigators were instructed to refer suspected severe AEFI cases to designated medical facilities. Data collection forms did not include name or date of birth of interviewed persons; instead, we assigned each data collection form a unique identifier. Data were kept on password protected servers and computers. Finally, when the decision was reached to publish findings, we requested a retrospective review by the Benin National Health Research Committee (CNERS); while the committee chairman indicated support for the study, he responded that the committee has no mechanism for retrospectively reviewing studies. A written response from the committee was provided to the Pan African Medical Journal.

**Comparison data:** we searched PubMed for citations from 2005-present using the terms (“MenAfriVac” OR “serogroup A conjugate vaccine”) AND (“safety”), which led to 125 results whose titles were screened for potentially relevant articles. These were reduced to eight by selecting those articles that indicated MenAfriVac had been evaluated. For these eight, we obtained the full manuscript and searched references of identified articles, searched additional articles by the lead authors of each manuscript, and reviewed citations identified in the PubMed tool “related citations in PubMed”. This led to two articles on MenAfriVac safety in Indian subjects [8, 17], three in African subjects [9, 14, 15], and two reports from the WHO Global Advisory Committee on Vaccine Safety [12, 13], plus the product package insert [7]. Because of the small number of trials, all were reviewed and used for comparison and identification of potentially important AEFIs; however, our results were compared formally only to data from the definitive licensing trials [8, 9].

**Evaluation design:** the Benin MoH elected to assign one district to receive MenAfriVac^®^ through a CTC approach during the already scheduled national mass immunization campaigns. As part of this initiative, the MoH wanted to document whether the CTC approach led to no greater occurrence of AEFIs that the traditional cold chain approach. Within this overall goal, the MoH and AMP agreed on the following specific objectives (1) to assess in the CTC district and a comparison district incidence rates of specific clinical symptoms or endpoints that had been reported in previous MenAfriVac^®^ vaccine AEFI studies; (2) to compare incidence rates with data reported in the literature. To achieve these objectives, we evaluated four villages in the district assigned by the Benin MoH to receive the CTC approach (Banikoara) and four villages in the comparison district (Kandi). The Benin MoH purposefully selected the two districts and eight villages based on socio-demographic aspects and feasibility. In particular, the very short timeline between the decision to conduct the evaluation and campaign initiation precluded randomisation and placed a premium on working in sites with sufficient staff and infrastructure. To obtain reasonably representative data, and given resource limitations that prevented evaluation of all vaccine recipients, we planned to identify participants randomly. However, identified villages did not have readily accessible databases of residents (e.g., census data, accurate housing maps, or phone directories). Consequently, in each village, households were selected by spinning a bottle in the village centre, starting investigation with the first household found in the direction indicated by the bottle, and then proceeding to the next closest household following that same direction until we had included the desired number of persons. All eligible persons (see below) were included from each selected household. A CTC approach meant that unopened vaccine vials could be kept at temperatures of up to 40°C for up to four days before injection. However, as an evaluation of a public health intervention under actual conditions encountered in the field, vaccine exposure duration to ambient temperatures was not standardized or mandated. Rather, a secondary objective was to assess actual ambient temperature exposure that occurred during a mass campaign. Residents in the comparison district received vaccine via the regular approach, i.e., kept in the +2 to +8°C temperature range throughout the delivery chain until the moment of the injection.

Evaluated persons were those who lived in a household in one of the designated villages; were present in the dwelling at the moment of the investigators’ visit; routinely slept in the household; had a vaccination card verifying vaccine receipt; and indicated willingness to participate in the survey. The target population for MenAfriVac^®^ is persons 1 to 29 years of age. However, we did not have any age restriction for our evaluation because during an actual campaign, vaccine might be delivered outside of target age groups. Evaluated AEFIs were those described in the MenAfriVac^®^ package insert [7] and others identified during previous pharmacovigilance studies during mass vaccination campaigns [14, 15]. The following data were collected via individual interviews of participants (or caretakers for small children) and a succinct clinical examination: socio-demographic information; symptoms; health care utilisation; vaccine lot number; axillary temperature and injection site examination; and history of hospitalisation or death of vaccinated household members who were not present at the time of the interview.

Household survey data were collected on five consecutive days starting the day following the vaccination day for villages where the campaign lasted a single day, and two days after the first vaccination day in villages where the campaign lasted several days. We included only a 5-day evaluation period because studies have found no evidence for AEFIs beyond 4 days after vaccination. Additionally, five days limited the possibility of recall bias. Finally, this allowed us to evaluate obvious clinical evidence of AEFIs at each of the first five days post immunisation. At each interview regardless of days post-vaccination and for each question we considered the full period between the moment of vaccination and the time of the interview. Because local informants indicated it was unlikely that we would find the same people at home on each day, we decided to choose participants randomly each day rather than follow a cohort over time. Investigators indicated that of the 630 households visited during the evaluation, 86 (14%) were visited more than once during the 5-day survey duration. Because of the relatively small percentage of reinclusion, we ignored this issue when calculating person day denominators for incidence rate estimations.

Temperature monitoring was performed to ensure that comparison and CTC vaccine remained within the target temperature range. Temperature monitoring was conducted with electronic thermometers while the vaccines were kept in refrigerators and afterwards with vaccine vial monitors placed on the vaccine label. For the CTC group an additional peak threshold indicator was placed into each of the vaccine carriers that were removed from the traditional cold chain. These stickers would change colour when exposed to temperatures exceeding 40°C, which if it occurred would result in discarding the vaccine vials. Eight clinical investigators were recruited and trained to conduct the field part of the evaluation including seven nurses and one health technician, supervised by an epidemiologist. Each investigator was assigned to a single village for the duration of the field assessment. The questionnaire was in French and questions were translated orally in the local language.

**Power calculations:** as indicated above, our evaluation was designed to evaluate AEFIs associated with public health vaccine use, and was limited by the time constraints of MenAfriVac^®^ roll-out and limited finances. Nevertheless, power calculations were performed to assess the degree of effects we could detect. Because the number of villages was fixed a priori, we estimated that to have 80% power to detect an increase in 5-day incidence of any particular AEFI from 3% to 6% at the 95% confidence level, we would need an to include 750 people per vaccine delivery method (CTC or non-CTC). To account for unforeseen effects, and because of a small marginal increase in cost, we increased this to 1000 people per vaccine delivery method or approximately 3000 person-days of observation based on enrolling 200 persons during each of the five days.

**Data management and analysis:** data from paper records were double-entered into an electronic database developed with Epidata^®^ software. Data were analysed with Stata Intercooled 11^®^ software package. Incidence rate ratio confidence intervals were calculated with a Poisson model, proportions were compared with the chi-square test, and continuous variables were compared with the Student t-test. All statistical tests were two-sided with a cut-off of 0.05 for statistical significance.

**Role of the funder:** the funder provided technical input into the study interpretation and draft manuscript but had no editorial control over the final manuscript contents. Christoph Steffen had full access to the study data and along with the Benin MoH made the final decision to submit for publication.

## Results

As measured at the Kandi weather station, temperatures ranged from 19 to 46°C during the vaccination period. A total of 1000 participants were included in the CTC and 999 in the non-CTC group. The CTC group used lot number 127M1045 and the non-CTC group lot number 127M1047. Details on site characteristics are found in Table 1. Eighteen persons had missing information for sex, and nine had missing information for age. For pregnancy and breastfeeding status, respectively, 543 and 539 women had missing values because investigators assumed wrongly that only positive status should be reported. We assumed in our analysis that all women where information on pregnancy or breast-feeding status was missing, were not pregnant or not breast-feeding (Table 2). The total person-days of follow-up was 3140 in the CTC and 2818 in the non-CTC group. The mean duration between immunisation and interview was 3.1 days in the CTC group and 2.8 days in the non-CTC group (p < 0.05) (Table 3). Age and sex distribution did not differ between CTC and non-CTC groups; all participants were 1-29 years of age, indicating we did not identify persons that received vaccine outside of the target age group. In the non-CTC group, data provided by the MoH confirmed that vaccines were kept in the cold chain throughout the vaccination campaign. The cold chain consisted of refrigerators with regular temperature monitoring until the day of vaccination and cold boxes with ice packs until the moment of the injection. Vaccine vial monitors were checked before use.

**Table 1:** Overview of villages that delivered MenAfriVac^®^ with and without a controlled temperature chain (CTC), Benin 2012

	Banikoara (CTC group)					Kandi (Control group)				
Village	Atabenou	Bonni	Sonwari	Kanderou	Summary	Kassakou	Donwari	Tankongou	Thya	Summary
Health centre providing the vaccine	Toura	Goumori	Kokey	Founougo		Kassakou	Donwari	Sam	Angaradebou	
Distance to urban centre	6 km	42 km	49 km	50 km	6-50 km	6 km	20 km	42 km	35 km	6-42 km
Estimated population in 2011	5437	4430	10144	4960	24971	5388	7454	5037	4297	22176
Vaccination date(s) in November 2012	16	18	17-18	15-17	15-18	15-18	15-18	15-17	15-19	15-19
Study period in November 2012	17-21	19-23	18-22	17-21	17-23	17-21	17-21	17-21	17-21	17-21
Number of subjects included	251	250	250	249	1000	249	250	250	250	999
Number of households visited	49	48	75	54	226	98	82	106	118	404
Average number of inclusion per household	5,1	5,2	3,3	4,6	4,4	2,6	3,1	2,4	2,1	2,5
Number of households sampled more than once during the 5 days	2	3	5	10	20	1	30	15	20	66
Number of subjects who refused to participate	3	0	0	0	3	0	0	2	0	2
Number of subjects excluded for not showing vaccination card	15	0	0	30	45	2	57	1	34	94

**Table 2:** Comparison of mean age, sex ratio, pregnancy and breast-feeding prevalence rates between MenAfriVac^®^ as used with and without a controlled temperature chain (CTC), Benin 2012

	CTC (n)	No CTC (n)	p-value for difference between CTC and no CTC groups
Mean age (in years)	10·0 (993)	10·1 (997)	0·55
Sex ratio M/F	0·63	0·74	0·072
Pregnant women*	3·2% (18/566)	4·6% (28/613)	0·22
Breast-feeding women*	16·3% (92/566)	10·0% (61/613)	0·001
**Interval between vaccination and interview**			
1 day	17·5% (175)	12·7% (127)	<0·0001
2 days	20·1% (201)	34·8% (348)	
3 days	19·7% (197)	22·8% (228)	
4 days	23·5% (235)	16·9% (169)	
5 days	21·0% (210)	12·7% (127)	

* We assumed in our analysis that all women where information on pregnancy or breastfeeding status was missing, were not pregnant or not breastfeeding, as appropriate

**Table 3:** Incidence rates (IR) (per 1000 person days) and incidence rate ratios (IRR) with 95% confidence intervals (CI) for different adverse events following immunization (AEFIs) among persons receiving MenAfriVac^®^ delivered with and without a controlled temperature chain (CTC), Benin 2012

	CTC			Control			IRR (95% CI)
Signs and symptoms	Person days	IR	(n)	Person days	IR	(n)	
Reported symptoms							
Fever	3137	8·3	26	2813	36·6	103	0·23 (0·15 to 0·35)
Pain at injection site	3135	15·9	50	2818	55·0	155	0·29 (0·21 to 0·40)
Rash	3140	3·2	10	2818	7·5	21	0·43 (0·20 to 0·90)
Diarrhoea	3140	0·3	1	2818	3·9	11	0·08 (0·02 to 0·63)
Loss of appetite	3140	0·6	2	2818	2·8	8	0·22 (0·05 to 1·06)
Nausea/ vomiting	3140	1^a^6	5	2818	5·7	16	0·28 (0·10 to 0·77)
Irritability	3139	0	0	2816	1·4	4	-
Headache	3140	6·1	19	2818	11·7	33	0·52 (0·29 to 0·91)
Asthenia	3140	1·6	5	2818	11·4	32	0·16 (0·06 to 0·40)
Myalgia	3140	0·6	2	2816	9·6	27	0·07 (0·02 to 0·31)
Arthralgia	3140	0·3	1	2811	2·5	7	0·14 (0·05 to 0·36)
Personal history							
Consultation	3148	0	0	2799	1·4	4	-
Hospitalisation	3118	0	0	2758	0	0	-
Clinical examination							
Fever (> = 38°C)	3140	5·7	18	2818	9·6	27	0·60 (0·33 to 1·10)
Induration/swelling	3140	2·5	8	2794	6·4	18	0·36 (0·17 to 0·74)
Erythema	3135	1·3	4	2798	0·7	2	1·79 (0·33 to 9·74)
Heat at injection point	3131	0·6	2	2798	2·5	7	0·26 (0·05 to 1·23)
Pain at examination	3132	6·4	20	2798	23·8	67	0·27 (0·16 to 0·44)
Fluctuance	3135	0	0	2790	0·7	2	-
Severe case detection in household survey							
Cases of hospitalisation in household members			0		0	-	-
Cases of death in household members			0		0	-	-

None of the interviewed persons indicated that they or household members had experienced hospitalization or death. Four interviewees (three from Kassakou) had sought medical care between vaccination and interview day, all belonging to the non-CTC group. The reasons for medical consultation were: fever; fever and headache; vomiting; and vomiting and headache. None was hospitalised during the course of the evaluation. For all AEFIs under investigation, the incidence rates in the CTC group were no different or lower than those in the non-CTC group (Table 3). The incidence of certain symptoms varied considerably between villages even within villages that had the same CTC status. Kassakou, a non-CTC village, showed the highest incidence rates among villages for most AEFIs. For example, over two thirds of the reports of pain at injection site, over half of the reports of fever and almost all reports of asthenia came from Kassakou village (Table 4). To evaluate the impact of the Kassakou data on the overall incidence we performed a sub-analysis for all AEFIs excluding Kassakou village. Incidence rate ratios for all individual AEFIs became non-statistically significant except for reported fever (IRR = 0.41 (0.25-0.66)), and myalgia (IRR = 0.14 (0.03-0.64)).

**Table 4:** Symptom counts for different adverse events following immunization (AEFIs) among participants receiving MenAfriVac^®^ delivered with and without a controlled temperature chain (CTC), stratified by village, Benin 2012

Signs and symptoms	CTC group					Control group					Prevalence ratio (95% CI)
	Atabenou (n = 251)	Bonni (n = 250)	Sonwari (n = 250)	Kanderou (n = 249)	TOTAL CTC	Donwari (n = 250)	Kassakou (n = 249)	Tankongou (n = 250)	Thya (n = 250)	TOTAL Control	
**Self-reported**											
Fever	15	1	5	5	26	2	58	32	11	103	0.25 (0.17, 0.38)
Pain at injection site	29	8	8	5	50	0	108	40	7	155	0.32 (0.24, 0.44)
Rash	6	3	0	1	10	0	20	1	0	21	0.48 (0.23, 1.0)
Diarrhoea	0	0	1	0	1	0	11	0	0	11	0.091 (0.011, 0.70)
Loss of appetite	0	0	0	2	2	0	5	1	2	8	0.25 (0.053, 1.2)
Nausea/ vomiting	2	0	1	2	5	0	12	4	0	16	0.31 (0.15, 0.85)
Irritability	0	0	0	0	0	0	2	2	0	4	0 (0, undef)
Headache	11	1	1	6	19	1	25	6	1	33	0.58 (0.33, 1.0)
Asthenia	4	0	0	1	5	0	31	1	0	32	0.16 (0.061, 0.40)
Myalgia	2	0	0	0	2	0	17	9	1	27	0.074 (0.018, 0.31)
Arthralgia	1	0	0	0	1	0	6	1	0	7	0.14 (0.018, 1.2)
**Clinical examination**											
Fever (> = 38°C)	5	3	7	3	18	2	8	8	9	27	0.67 (0.37, 1.2)
Induration/ swelling	2	1	0	3	6	0	4	7	0	11	0.54 (0.20, 1.5)
Erythema	3	0	0	1	4	0	2	0	0	2	2.0 (0.37, 11)
Heat at injection point	1	0	0	1	2	0	2	5	0	7	0.29 (0.059, 1.4)
Pain	4	8	3	5	20	0	46	19	2	67	0.30 (0.18, 0.49)
Fluctuance	0	0	0	0	0	0	2	0	0	2	0 (0, undef)

In the CTC group, vaccine exposure to ambient temperature varied between 1 and 82 hours (mean duration 20 hours) before injection, depending on the day and the place where participants presented for vaccination and the time of vaccination (Table 5); among the 982 CTC vaccine recipients for whom this information was known, 655 (67%) received vaccine that had remained at ambient temperatures for no more than 12 hours. In Kanderou, the entire vaccine stock available for the village was removed from the cold chain on the morning of November 15^th^. Vaccination started on the 15^th^ and finished on the 17^th^. For Sonwari, vaccines were removed from the cold chain on November 15^th^ in the morning, and vaccination was implemented on November 17^th^ and 18^th^. In Atabenou and Bonni, vaccines were removed from the cold chain on the day of vaccination. The precise time period during which vaccine was delivered on a given day was not known. None of the vaccine vial monitors had changed color to indicate excessive heat exposure. We did not identify any relationship between the duration of vaccine exposure to ambient temperature before injection and the incidence of measured or reported fever (Table 5) or any other AEFIs under investigation (data not presented).

**Table 5:** Incidence rate ratios (IRR) and 95% confidence intervals (CI) of measured and reported fever among persons receiving MenAfriVac^®^ delivered with and without a controlled temperature chain (CTC), stratified by duration of vaccine exposure to ambient temperature, Benin, 2012

	Axillary température ≥ 38°C		Reported fever	
Exposure duration (hours)	n/N	IRR (95% CI)*	n/N	IRR (95% CI)*
< 1 (control group)	27/999	Ref.	103/998	Ref.
1-12	10/655	0·51 (0·25 to 1·05)	17/654	0·23 (0·14 to 0·38)
13-36	1/77	0·32 (0·04 to 2·35)	3/77	0·25 (0·08 to 0·79)
37-60	6/212	1·08 (0·44 to 2·61)	5/211	0·23 (0·10 to 0·58)
>60	1/38	0·80 (0·11 to 5·91)	0/38	0

* IRR and confidence interval are calculated using Poisson regression model adjusted for person-days

## Discussion

This is the first field-based evaluation of the CTC methodology during a public health immunisation campaign. The NmA conjugate vaccine MenAfriVac^®^ specifically has received licensure for this approach based on demonstration of vaccine stability, and no novel adverse events were expected. However, because unexpected events may occur, confirmation of this assumption was necessary. This is particularly true when implementing vaccine outside of a controlled study in a public health campaign. As a new vaccine, relatively limited post-licensure safety data are available for MenAfriVac^®^. We found no cases of hospitalisation or death, no increased occurrence of adverse events in persons receiving vaccine through the CTC versus the routine cold chain approach, and no increased incidence of adverse events in the CTC group compared to historical data for MenAfriVac^®^. These findings provide some initial support for the CTC approach and thus support the safety record of MenAfriVac^®^ 13. To compare more easily AEFI incidence rates with data from the literature, where AEFI incidence rates usually are presented as percentages over a four or seven day period, we compared a subset of our data corresponding to day 4 and day 5 cohorts to previous data from the literature [8, 9] or the MenAfriVac^®^ package insert [7] (Table 6), and again found favourable results for the CTC approach. The CTC approach allows for exposure to ambient temperatures for up to 4 days, or 96 hours. In our setting, the actual exposure duration usually was much shorter, with a mean of 20 hours, and none of the exposures exceeded the recommended limit even if this limit was approached. Additionally, we did not see evidence for increased fever or other outcomes as exposure duration increased. These data confirm the practicality of the CTC approach, including that the exposure duration limit in the package insert is likely to be sufficient to achieve objectives during a campaign setting.

**Table 6:** Comparison of adverse events following immunization (AEFI) incidence rates observed in the current study for persons receiving MenAfriVac^®^ delivered with and without a controlled temperature chain (CTC) for observations on day 4 or 5 post vaccination with incidence rates reported in the MenAfriVac^®^ vaccine package insert and the literature

AEFIs	CTC% (95%CI)	Control% (95%CI)	MenAfriVac^®^ package insert [7]%	Kshirsagar et al. 2007 [8]% (95% CI)	Sow et al. 2011 [9] % (95%CI)
(Follow-up time post immunisation)	4-5 days	4-5 days		7 days	4 days
Fever	1.1 (0.4-2.6)	8.1 (5.2-12.1)	2 to 7	0 (0-14)	3.0 (1.8-4.7)
Pain at injection site	1.6 (0.6-3.2)	7.8 (4.9-11.7)	2 to 30	75 (53-90)	5.0 (3.4-7.0)
Rash	0.4 (0.1-1.6)	0.7 (0.1-2.4)	-	13 (3-32)	
Diarrhoea	0	0.7 (0.1-2.4)	≤13	0 (0-14)	1.0 (0.4-2.1)
Loss of appetite	0	0.3 (0-1.9)	≤10		0.5 (0-2.1)
Nausea/ vomiting	0.4 (0.1-1.6)	1.0 (0.2-3.0)	≤10	0 (0-14)	1.3 (0.6-2.6)
Irritability	0	0.7 (0.1-2.4)	≤12		0 (0-1.8)
Headache	0.9 (0.2-2.3)	2.0 (0.7-4.4)	≤11	4 (0-21)	11.2 (8.3-14.7)
Asthenia	0.2 (0-1.3)	2.7 (1.2-5.3)	≤1	4 (0-21)	1.5 (0.6-3.2)
Myalgia	0	1.0 (0.2-3.0)	≤1		0.7 (0.2-2.2)
Arthralgia	0	0.3 (0-1.9)	≤1	4 (0-21)	0.5 (0.1-1.8)

**Limitations:** Our evaluation had several limitations related firstly to it being a public health intervention under real-life conditions rather than a study and secondly to its development and implementation within three weeks to meet the MenAfriVac^®^implementation schedule. Our evaluation was not randomised since the Benin MoH used a non-random method to select the evaluation districts and villages. Furthermore, spinning the bottle in the center of town also could have selected a non-representative population. Given the same quality vaccine, previous studies have found similar AEFIs among populations even across continents [7–9, 12–15]. Nevertheless, if our design selected areas in which vaccine handling and delivery, as well as environmental conditions, differed from non-selected areas, our results may not be representative of AEFIs following the CTC approach for the entire Benin meningitis belt population. For practical reasons, we included only persons present at the house during our visit; this could have led to an underestimation of AEFI prevalence if some persons were absent because they were having an AEFI evaluated.

As mentioned previously, vaccine exposure duration to ambient temperatures in CTC villages was not standardized or maximized, preventing a robust study of AEFIs at 96 hours of exposure. We did not assess rare events, events occurring after 5 days post-immunisation, or events not expected based on previous data. By report of field staff, proper immunisation practices were more emphasised in the CTC villages than non-CTC villages. We could not account for the possible effect of illness due to causes other than the vaccine because we did not have the ability to conduct thorough health examinations including diagnostic testing. Lastly, for practical reasons, we did not rotate on a daily basis field staff between CTC and other groups or villages. This might have resulted in systematic classification biases as these staff might have collected information in slightly different ways, particularly since they were not blinded to the CTC status in their village. This may be the reason why a single non-CTC village had greatly increased rates for most outcomes.

**Additional studies:** To allow CTC vaccines to accomplish their full potential, additional studies will be needed. Anthropological studies should be conducted to ensure that vaccinators and recipients do not consider vaccine delivered through the CTC approach to be inferior or damaged. Economic studies should be considered to assess benefits of the CTC approach within specific situations, such as routine immunisation or limited to campaigns. Logistical studies should assess the risk of the CTC approach on cold chain breaks for concurrently administered and more heat labile vaccines. Anthropological studies could define more clearly health work attitudes and behaviour related to implementation of differing cold chain standards for different studies. Finally, the limits of the CTC approach need to be more clearly defined, included maximum time-temperature exposure and reintegration into the cold chain of unused vaccine vials.

MenAfriVac^®^ is the first vaccine used in a public health immunisation programme in Sub-Saharan Africa that is licensed for temporary storage under ambient temperature. On a continent where temperatures peak above 30°C in many countries throughout the year, where transport infrastructure is still weak, and where sufficient resources for cold chain maintenance are lacking, the CTC approach can have a large impact. Specifically, the CTC approach could decrease cold chain expenses, minimize risks to scheduled immunization days from supply chain ruptures, make more efficient use of human resources, increase the number of vaccination sessions, and hence improve overall immunization coverage. While we acknowledge substantial methodological limitations, our evaluation provides initial evidence that MenAfriVac^®^ can be used safely on a large scale using the CTC approach, increasing the flexibility and efficiency of its use.

## Conclusion

Following use of the CTC approach with MenAfriVac^®^ in a public health immunization campaign, occurrence of clinical outcomes during the first 5 days following immunization was no higher than in a comparison district using the traditional cold chain approach or outcomes reported in previous clinical trial settings. This initial study supports use of MenAfriVac^®^ with the CTC approach but additional studies are needed before more routine roll-out.
